# A systematic bioinformatics approach for large-scale identification and characterization of host-pathogen shared sequences

**DOI:** 10.1186/s12864-021-07657-4

**Published:** 2021-09-28

**Authors:** Stephen Among James, Hui San Ong, Ranjeev Hari, Asif M. Khan

**Affiliations:** 1 Centre for Bioinformatics, School of Data Sciences, Perdana University, Damansara Heights, Kuala Lumpur 50490 Malaysia; 2 Department of Biochemistry, Faculty of Science, Kaduna State University, Kaduna, 800211 Nigeria; 3 Beykoz Institute of Life Sciences and Biotechnology, Bezmialem Vakif University, Beykoz, Istanbul, 34820 Turkey

**Keywords:** Shared sequences, Share-ome, Host-pathogen, Bioinformatics, Large-scale, Methodology, *Flaviviridae*, *Flavivirus*, *Hepacivirus*, *Pegivirus*, *Pestivirus*, *Dengue virus*, *West Nile virus*, *Hepatitis C virus*, Cross-reactivity, Crossreactome, Peptide sharing, Peptide overlap, and Molecular mimicry.

## Abstract

**Background:**

Biology has entered the era of big data with the advent of high-throughput omics technologies. Biological databases provide public access to petabytes of data and information facilitating knowledge discovery. Over the years, sequence data of pathogens has seen a large increase in the number of records, given the relatively small genome size and their important role as infectious and symbiotic agents. Humans are host to numerous pathogenic diseases, such as that by viruses, many of which are responsible for high mortality and morbidity. The interaction between pathogens and humans over the evolutionary history has resulted in sharing of sequences, with important biological and evolutionary implications.

**Results:**

This study describes a large-scale, systematic bioinformatics approach for identification and characterization of shared sequences between the host and pathogen. An application of the approach is demonstrated through identification and characterization of the *Flaviviridae*-human share-ome. A total of 2430 nonamers represented the *Flaviviridae*-human share-ome with 100% identity. Although the share-ome represented a small fraction of the repertoire of *Flaviviridae* (~ 0.12%) and human (~ 0.013%) non-redundant nonamers, the 2430 shared nonamers mapped to 16,946 *Flaviviridae* and 7506 human non-redundant protein sequences. The shared nonamer sequences mapped to 125 species of *Flaviviridae*, including several with unclassified genus. The majority (~ 68%) of the shared sequences mapped to Hepacivirus C species; West Nile, dengue and Zika viruses of the *Flavivirus* genus accounted for ~ 11%, ~ 7%, and ~ 3%, respectively, of the *Flaviviridae* protein sequences (16,946) mapped by the share-ome. Further characterization of the share-ome provided important structural-functional insights to *Flaviviridae*-human interactions.

**Conclusion:**

Mapping of the host-pathogen share-ome has important implications for the design of vaccines and drugs, diagnostics, disease surveillance and the discovery of unknown, potential host-pathogen interactions. The generic workflow presented herein is potentially applicable to a variety of pathogens, such as of viral, bacterial or parasitic origin.

**Supplementary Information:**

The online version contains supplementary material available at 10.1186/s12864-021-07657-4.

## Background

There has been an exponential growth of pathogen sequence data, given their relatively small genome size and important role as infectious and symbiotic agents [1–5]. This has largely been driven by high-throughput omics technologies, resulting in petabytes of data proliferating publicly available databases, providing access for knowledge discovery and study of the complex molecular descriptors of host-pathogen interactions [6, 7]. This enables detection of patterns for disease tracking and surveillance, control of pathogens, and clinical prognosis of infectious diseases, which are useful in monitoring and forecasting emerging pathogens [8, 9].

Pathogens, such as viruses, bacteria and parasites infect a wide range of hosts, such as human, farm/domestic animals and plants, and are responsible for high mortality, morbidity and/or damage [10, 11]. Pathogen sequences integrated into the host genome is not uncommon [12–15] and evolutionary sharing of sequence with the host has also been reported [16, 17]. A shared sequence is one where part or full-length of a pathogen protein is shared with one or more protein sequences of the host [18]. A pathogen sequence integrated into the host genome is herein considered as part of the host proteome, if expressed. Thus, shared sequences can be a result of pathogen integration into the host or otherwise. For example, the footprint of viruses in the evolution of the mammalian genome is thought to go back to at least tens of millions of years, in contrast to the earlier estimate of a few thousand. Taylor et al. (2010) confirmed that several groups of mammals, including marsupials that never colonized Africa, have had an association with filoviruses. This discovery of shared sequences between host-pathogen has important implications for the design of vaccines and drugs, diagnostics, disease surveillance and the study of emerging diseases, including unknown, potential host-pathogen interactions [19].

Shared sequences have been implicated in various cellular processes, which includes signalling, transduction, and protein stability [20–22]. These processes have been described to play a key role in pathogenicity of the host. Earlier studies of shared sequences or molecular mimicry have been based on similarity search for sequences of *k-mer* lengths of mostly penta-, hexa-, hepta-, or octapeptide (5-, 6-, 7- or 8-mer), and generally applied on a limited number of sequences of the pathogen of choice, such as *Human gammaherpesvirus 4* (*Epstein-Barr virus*) [23], *Human cytomegalovirus* (HCMV) [24], *Human immunodeficiency virus 1* (HIV-1) [18, 21], *Poliovirus* [25], *West Nile virus* (WNV) [22], *Measles virus* [26], *Influenza A virus* [16], *Streptococcus* species [27], *Mycobacterium tuberculosis, Salmonella typhimurium, Klebsiella pneumonia*, and *Proteus mirabilis* [28], among others. The availability of large data in the public repositories means much remains to be elucidated on shared sequences, which can further broaden our understanding of host-pathogen interactions [29, 30].

Existing alignment-based computational tools can be utilised for similarity search between host and pathogen sequences, such as BLAST [31], FASTA [32], and SSEARCH, among others. Limitations of these tools include i) the non-exhaustive nature of the search given the heuristic approach, which means not all *k-mers* may be compared to each other exhaustively, ii) gaps introduced in the alignment may break the collinearity of *k-mers*, iii) restriction on the number of hits returned (such as 20,000 for BLAST), iv) restriction on the number of queries per batch submission, and iv) time delay due to the iterations required to deal with the issues of (iii) and (iv) through a “break and conquer” approach, to deal with the large number of host and pathogen sequences available.

Herein, we describe a systematic bioinformatics approach for identification and characterization of shared sequences from big data. The approach is generic, and thus is potentially applicable to any pathogen and host combinations. A large number of protein sequences are available for pathogens and hosts in public repositories. The complete set of the identified shared sequences for a given host-pathogen will be termed as the share-ome. The identification, characterization and comparative analysis of multiple host-pathogen share-omes has important implications in the understanding of the evolution, structure and function of shared sequences.

## Materials and methods

The relevant bioinformatics tools, web servers, and tutorials described herein are collectively listed in Table 1 with the corresponding URLs.

**Table 1:** Tools, databases and tutorials relevant for the identification and characterization of the host-pathogen share-ome. All URLs were accessible as of January 2021

Database, Tool, and Tutorial	URL
NCBI Entrez Databases	http://www.ncbi.nlm.nih.gov
NCBI Entrez Protein Database	http://www.ncbi.nlm.nih.gov/protein
NCBI Entrez Taxonomy Database	http://www.ncbi.nlm.nih.gov/taxonomy
STRING Viruses database	http://viruses.string-db.org/; version 11.0
CD-HIT	http://weizhongli-lab.org/cd-hit/
E-Utilities esearch-efetch	https://www.ncbi.nlm.nih.gov/books/NBK25500/#chapter1.Demonstration_Programs
entrezEsearch	https://github.com/gwatiyapJ/SiMiLyG
*kmerslicer*	https://github.com/gwatiyapJ/SiMiLyG
Unipro UGENE tools	http://ugene.net/
UniProt Retrieving and ID Mapping tool	https://www.uniprot.org/uploadlists/
CateGorizer	https://www.animalgenome.org/tools/catego/
Unix utilities	https://unix.stackexchange.com/
Tutorial 1: Notes on how to use R for doing statistical analysis and graphics	https://cran.r-project.org/manuals.html
Tutorial 2: Unipro UGENE Manual Version 37, 2020. Consists of user guide to bioinformatics tools for alignments, genome sequencing, data analysis, and amino acids sequence visualization, among others.	http://ugene.net/downloads/UniproUGENE_UserManual.pdf
Tutorial 3: User’s guides on implementation for removing duplicates sequences and generating representative sequences	http://www.bioinformatics.org/cd-hit/cd-hit-user-guide.pdf
Tutorial 4: Webinar: Introduction to NCBI’s E-utilities API	https://www.youtube.com/watch?v=iCFVVexp30o&t=2561s

**Fig. 1:**
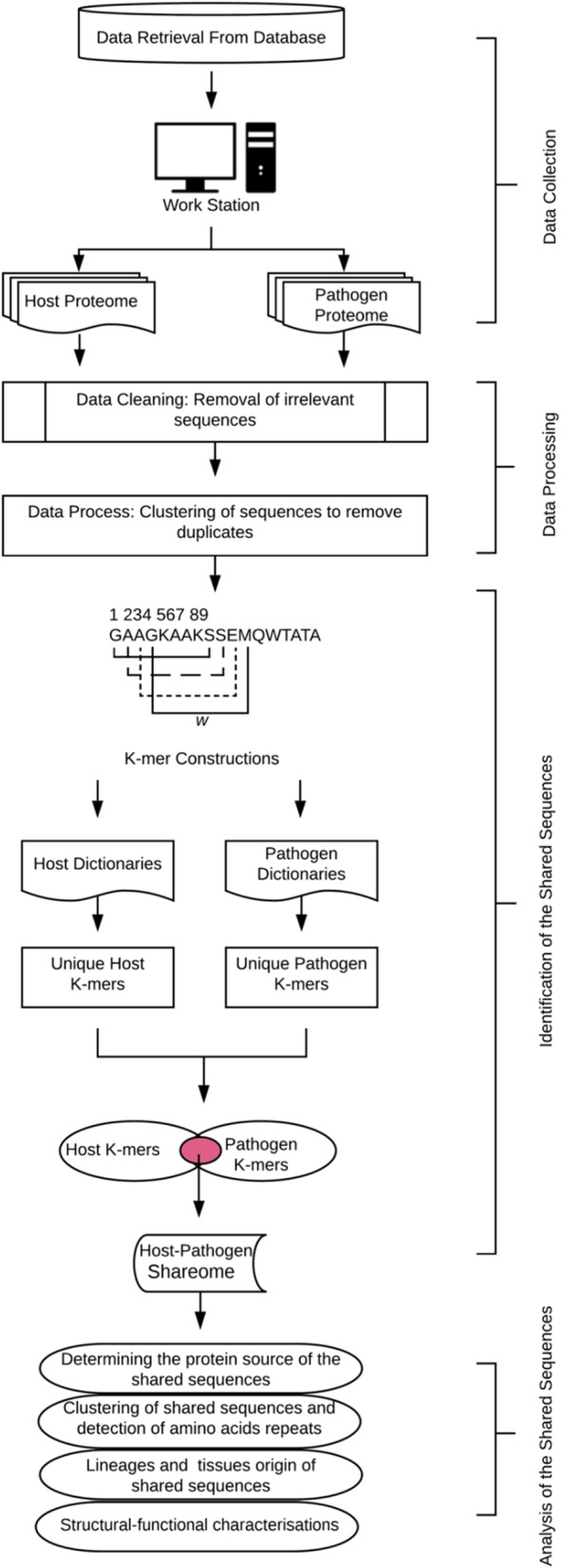
A schematic workflow for large-scale identification and characterization of host-pathogen shared sequences

A systematic bioinformatics approach is required to handle big data and mine for biological patterns and insights. The approach defined herein for the identification of the share-ome is a workflow that can be divided into four parts (Fig. 1): i) data collection, ii) data processing, iii) identification of the share-ome, and iv) share-ome analyses.

### Data collection

Both nucleotide and protein sequences, available in abundance in public repositories (Table 2), are essential for the study of structure, function and evolution of shared sequences. Thus, a collection of all reported primary sequences is necessary for a comprehensive survey of the distribution and composition of shared sequences. The National Centre for Biotechnology Information (NCBI) (Table 1) Entrez Nucleotide (nt) and Protein (nr) databases provide a comprehensive collection of primary sequences [33]. When taxonomy is a selection criterion (such as all viruses), the NCBI Taxonomy database (Table 1) [33] is ideal for filtering of sequences at various ranks of taxonomy lineage. The number of sequences can grow exponentially when navigating from species to higher ranks of taxonomy in the database, such as from species, genus, family, superfamily, order, class, phylum, to superkingdom. As such, download via a Hypertext Transfer Protocol (HTTP) option will not be appropriate and maybe terminated due to time-out issues, while a File Transfer Protocol (FTP) browsing offers limited functionality to restrict the data to an Entrez query term. Thus, the NCBI application programme interface (API), Entrez E-utilities is ideal for retrieval of large datasets. Although nucleotide sequences are important for the study of shared sequences, the focus herein is on protein sequences, which can be compared with the cognate nucleotide sequences subsequently for underlying synonymous substitutions. Nonetheless, the approach for analysis of nucleotide sequences would be similar to that of proteins.

**Table 2:** Human pathogens/parasites and big data. The available number of nucleotide and protein sequence records at NCBI Entrez Databases (as of January 2021) are indicated for select groups of pathogens/parasites. It should be noted that not all the species that are part of the taxonomic groups listed here maybe pathogens/parasites of human

Pathogen/Parasite	Sequence Data (# Records)	
	Nucleotides	Proteins
Viruses	3,554,899	7,360,073
Bacteria	68,010,589	773,862,087
Archaea	914,730	6,836,105
Fungi	13,642,359	25,043,777
Plasmodium	567,181	662,147
Amoebozoa	802,451	317,693
Trichomonas	245,291	121,145
Trypanosoma	425,666	408,483
Platyhelminthes (Flatworm)	3,326,558	787,530
Nematodes	4,321,847	1,776,489
Acanthocephalans	8863	2669
Hirudinea (Leeches)	255,634	52,331

### Data cleaning and processing

Removal of redundant and irrelevant sequences (including unknown, ambiguous, or outliers) is necessary to remove confounders in the identification of shared sequences. Public repositories often contain discrepancies and duplicate data entries [34–36]. Often, these are detected during the analysis step, such as when analysing the output of a multiple sequence alignment. A later detection of such sequences may necessitate a repeat of earlier data cleaning and pre-processing steps; thus, it is best to detect and filter these at the earliest. Examples of irrelevant sequences detected from supposedly a data of all viruses (Taxonomy Database ID: 10239) are shown in Table 3. The irrelevant hits may, for example, consist of sequences with no complete lineage information (i.e. no assigned viral species rank in the metadata, such as, only family rank), of unknown sample organism, that are synthetic construct or chimeric fusion protein, and/or of bacterial origin, mis-classified as viral or human data. One way of detecting these outliers at the data processing stage would be to extract the lineage field information in the metadata of each record and analyse for anomalies. Duplicate full-length or partial sequences can be filtered with the aid of the clustering tool, CD-HIT, which is capable of handling large datasets [37, 38].

**Table 3:** A sample of irrelevant hits identified through the data cleaning process. The irrelevant hits from a viral lineage search included those of missing/incomplete lineage information (unknown, unclassified or no species information) or of unrelated lineage (bacterial species) origin

Protein ID	“Organism” field value	Remark
CAA41747.1	*Retroviridae*	Species information not available
CAA41748.1	*Retroviridae*	“
AAB29320.1	*Tobamovirus*	“
AAB22506.1	*Orthohantavirus*	“
CAM83964.1	unclassified Parechovirus	“
5AUM_D	*Potyviridae*	“
2MLG_A	*Fuselloviridae*	“
1BDE_A	Unknown	“
1Q3Z_A	Unknown	“
3F2E_A	*Rudivirus*	“
ANM47321.1	*Streptococcus suis*	Bacterial origin
ANM47427.1	*Streptococcus suis*	“
AAT65035.1	*Mycoplasma fermentans*	“
AAT65057.1	*Mycoplasma fermentans*	“
BAA94190.1	*Escherichia coli O157:H7*	“
CAC83125.1	*Escherichia coli*	“
CAH23236.1	*Escherichia coli*	“
CAH23267.1	*Escherichia coli*	“
CAH23268.1	*Escherichia coli*	“
4PJZ_A	*Actinoplanes teichomyceticus*	“
4PK0_A	*Actinoplanes teichomyceticus*	“

Large-scale proteomics analyses usually present sample bias due to inadvertent collection of duplicate (full-length or partial identical match) or highly similar sequences, isolated from various geographical areas of limited catchment [39]. Removal of duplicate or similar sequences help, but may not be ideal to mitigate the bias. The redundancy may be a reflection of pathogen incidence in the ecosystem. Thus, it may be desired to analyse both the redundant and non-redundant datasets in most cases. However, for share-ome mapping and analysis, non-redundant dataset is preferred, with meta-data annotations from the redundant dataset retained. The annotations are retained because it is possible that, although the sequences are duplicates, the descriptions of the proteins are different. This maybe a result of user-dependent annotation (such as one description more detailed than the other) or are simply different protein names with the same sequence (related viral species sub-groups sharing the same sequence).

### Identification of a share-ome

#### Construction of a k-mer dictionary

*K-mers* are sequences of length *k* that can comprise of either nucleotides or amino acids. Detection of shared sequences, as matched pairs between host and pathogen, involves exhaustive *k-mer* matching at a certain identity or similarity threshold. A sliding window approach to generate a *k-mers* dictionary (i.e. total repertoire of distinct *k-mers* for each of the pathogen and host datasets) can reveal the complexity of sequences [40], provide a varied classifier for the sequence by capturing several neighbouring residues [41], and describe short structural elements and functional group positions [42]. Although the size of the *k-mer* is user specified, there are, however, certain recommendations for the search of shared sequences. The BLAST algorithm uses short “words” to nucleate regions of similarity and the default size for a protein sequence is three amino acid residues, while size of 11 bases is the default for nucleotide sequences [43]. The *k-mer* size of one or two residues is inapplicable as they would result in random matches. The significance of the pair match would increase as *k-mer* size increases. The human immune system recognises peptides of length of 8–12 residues for binding to human leukocyte antigen (HLA) class I molecules [44, 45] and 13–25 residues for class II, with nine being the typical length for class I and the binding core of class II peptides [45, 46]. As such, it could be argued that the immune system is able to effectively discriminate between “self” and “non-self” at this length (9-mer). Although viruses represent “non-self”, the shared sequences are a representation of “self” within “non-self”, with consequences that typically benefit the virus (e.g. as amino acid repeats or decoys) and/or harm the host (e.g. through autoimmune reaction). The immune system may not be well adept in discriminating the so-called “self” within the “non-self” from “itself”. Nonetheless, 9-mers appear to be the length of choice for antigen recognition over the evolutionary history of the adaptive (cellular) immune system in its defence against myriad of pathogens; indicating the need for a delicate balance between sensitivity and specificity.

The maximum *k-mer* size, if desired, can be the length of a given pathogen protein sequence of interest. It is noted that the longest consecutive, overlapping, identical *k-mer* match between viruses and human protein sequences can be in the range of hundredth amino acids, and this may expand to a longer length (possibly up to the protein size), particularly if one considers sequence similarity (using appropriate substitution matrix) rather than identity as the cut-off threshold for the match. Notably, the *cytomegalovirus* (CMV) virus codes for a protein that mimics the HLA molecule as a decoy [24].

A possible caveat of using longer *k-mers* is the likelihood of missing out shorter shared sequences. For example, a *k-mer* of nine (nonamer) would miss-out the detection of a shorter shared sequence, such as of six amino acids (6-mer), if the search criteria is a 100% match for the entire length of the 9-mer, and if the amino acids before or after the 6-mer (within the 9-mer region) are dissimilar. This can be circumvented by using a similarity search approach instead. Thus, in general, shorter *k-mers* of reasonable lengths provide for a higher sensitivity coverage of shared sequences, while the overlaps between them would encompass the longer higher specificity *k-mers*.

One can generate *k-mers* for a given input FASTA file using our in-house tool “*kmerslicer*” (Table 1). The tool can accommodate any defined *k-mer* window size and annotates each *k-mer* with meta-data, such as the accession number(s) of the origin sequence(s) from which the *k-mer* was generated, and the beginning position of the *k-mer* in the sequence. A *k-mer* dictionary is generated for each of the host and the pathogen datasets, and duplicate k-mers are removed (however, meta-data annotations are retained) to reveal the distinct *k-mers* for each. The two *k-mer* dictionaries can be collectively referred to as a host-pathogen library, which can be mined for shared sequences; a library may comprise of multiple dictionaries of different *k-mer* lengths for the respective host and pathogen.

#### Mapping of shared sequences

Matching *k-mers* between the corresponding *k-mer* dictionaries of a given host-pathogen library is key to identifying shared sequences. This matching has to be done at a certain sequence identity or similarity threshold. The most stringent threshold would be 100% identity, where identical *k-mers* between the dictionaries are matched. The longer the *k-mer* length, the lesser the number of expected match hits. The more stringent the threshold, the more significant will be the shared sequences, in particular for longer *k-mers*. Thresholds of identity lower than 100% allow for inclusion of nucleotide or amino acid variations (outcome of mutation or recombination, for example) within the *k-mer*. Setting thresholds by use of identity are more stringent than similarity. Nonetheless, similarity thresholds are ideal to identify shared sequences that may no longer be identical, but conserved physico-chemically. This would help capture shared sequences that may have evolved since the point of integration, such as HIV-1 motifs [12], or are analogs of similar structure, such as a CMV protein (UL18), analogous to HLA [24].

### Share-ome analyses

#### Determining the protein source of the shared sequences

Tracing the protein source of the shared sequences enables structure-function analysis of the share-ome. A given shared sequence can originate from multiple different protein sequences. The protein accession number is readily provided by the *kmerslicer* tool as the metadata information is stored alongside the *k-mer* sequence in the *k-mer* dictionary. The shared sequences and the corresponding protein accession data can be stratified to glean for information such as i) most abundant shared sequence (present in the most number of different proteins); ii) the least abundant shared sequence; it is possible that for specific host-pathogen relationships, the least abundant shared sequence could be in more than one protein; iii) pathogen species origin of the proteins containing the shared sequences (single or multi-species).

#### Clustering of shared sequences and detection of amino acid repeats

Proteins of host and pathogen can be highly represented and packed with shared sequences. Shared sequence representation (SSR) for a given protein is the fraction of the proteome-wide identified shared sequences that are present in the protein; i.e. the number of shared sequences present in a protein divided by the total number of shared sequences identified for the proteome, converted as a percentage:

$$ SSR(p)=\left(\frac{n(p)}{n(S)}\right)\times 100 $$

where *n* is the number of shared sequences present in a protein of interest, *p*, or the proteome, *S*.

Shared sequence packing (SSP) for a given protein is the length of the protein spanned by the identified shared sequences over the total length of the protein; i.e. the total contiguous length of the shared sequences present in a protein divided by the total length of the protein, converted as a percentage:

$$ SSP(p)=\left(\frac{\sum \limits_{i=1}^{n(p)}{l}_{i,p}}{L_p}\right)\times 100 $$

where *i* is a given shared sequence, with *n* as the number of shared sequences present in a protein of interest, *p*, and *l* is the contiguous length of the shared sequences, while *L* is the length of the protein, *p*. For example, a given viral protein, *p* of length 100 amino acids and containing three nonamer shared sequences (spanning a contiguous length of 11 amino acids), originating from a viral proteome, *S* comprising of 2537 nonamer shared sequences (share-ome), would have an SSR of ~ 0.12% and an SSP of ~ 11%. It should be noted that when the shared sequences map to multiple contiguous regions in a protein, the sum of the lengths of these regions should be used for the SSP calculation. An SSP of 100% for a protein would indicate that the full-length of the protein is shared, while an SSR of 100% for a protein would indicate that all the shared sequences of the proteome originated from the given single protein.

Shared sequences can also harbour amino acid repeats (AARs). These repeats can be either covering a subset or the full-length of the shared sequence. In terms of complexity, the repeats can be either simple (low-complexity) or complex; and in terms of distance, they can be tandem or non-tandem repeats. Identification of AARs can be carried out by use of the Dotplot plugin in the Unipro UGENE toolkit [47], which can handle big data. The Dotplot plugin provides a means for self- or non-self-comparison between two sequences, allowing identification and visualization, at gross level, of structural features of alignments, such as direct and inverted repeats (including palindromes), besides mutations, inversions, insertions, and deletions. A help webpage on how to use the Dotplot plugin is provided (URL in Table 1). AARs may represent important functional or structural motifs or domains, that can provide insight or footprint from molecular sequence on evolutionary mechanisms of host-pathogen interactions [48].

Proteins can also be littered with clusters of shared sequences (hotspots), which can be defined as regions in the protein that contain multiple (at least three or more) shared sequences, overlapping by at least one amino acid. The clusters can be of a minimum length of 11 amino acids to a significantly large size, such as >100aa. Such hotspots possibly represent regions of high host-pathogen interaction activity and may be of important structural or functional implications. Presence of AARs within these hotspots can strengthen this assertion further [48].

AARs and hotspots are expected to exist in share-ome containing protein sequences, and AARs have been reported to play a key role in protein structure and function [49], such as molecular recognition and molecular assembly [48]. Additionally, abundance of repeats may be an indication of selective pressure exerted on the genome, signifying a conserved region among orthologous proteins, as observed in lysine, glutamic acid, proline, serine and alanine rich repeat proteins [50].

#### Lineages and gene ontologies of shared sequences

Determining the organismal lineage origin of the pathogen proteins that contain the shared sequences can reveal the identity of the pathogen species contributing to the share-ome; this information may help better understand the significance of the shared sequence and may provide relevant insights and/or implications of the pathogen to the host, in particular if no prior or limited interactions have been reported. The lineage information can be used to further characterize the pathogen shared sequences in terms of structural/functional comparisons, such as providing insights on homology and virulence factors, which can aid in understanding of pathogen mechanism of action [51], and/or reveal evolutionary trajectories of the pathogen variants and their replication mechanisms [52, 53]. Separately, analysis of gene ontologies of shared sequences is important to understand how and where the effect of the shared sequences maybe exerted. The gene ontologies can be studied for the host proteome (and for specific groups of pathogens, if gene ontology terms are well-established) through an enrichment analysis for cellular component by use of the Gene Ontology (GO) sever [54]. Additional characterization maybe performed on the identified shared sequences to better understand their function and structure, in particular host-defence implications.

#### Functional-structural characterizations

Numerous tools are available to develop bioinformatics approaches for detailed characterization of a mapped share-ome, in terms of structure and function. Approaches for functional characterization of pathogen and human molecular sequences have been described [55], facilitated by various tools that allow search for known functions reported in biological databases; and/or prediction of putative functions, modelled using various methods. Such prediction resources include protein family database (Pfam) [56], InterPro [57], conserved domain database (CDD) [58], and GO terms for enrichment analysis [59], among others. A specific aspect of function is the assessment of immunological relevance, given the autoimmune implications of the shared sequences. Both cellular and humoral immune responses are applicable to pathogen sequences. Humoral, B-cell epitopes can be predicted by use of tools [60, 61], such as BcePred, BEST, Pep-3D-Search, PepSurf, CED and PEPITO/BEPro, among others. Similarly, T-cell epitopes can be predicted by use of tools, such as CTLPred and NetCTLpan [60], among others.

Structural characterization may involve determining the 3D localisation of the shared sequence in the cognate protein [62] by use of a structure visualisation tool (such as VMD [63]), and assessing its surface accessibility and secondary structure by use of NetSurfP [64] or PDBePISA [65] and DSSP [66], respectively. Homology models may be built when a 3D structure is not available, either manually using Modeller or in an automated-fashion using Swiss-Model. Docking and 3D simulation maybe carried out to determine the binding efficacy of ligands using a number of tools available, such as AutoDock [67] and Gromacs [68].

## Results

### Application: identification and characterization of *Flaviviridae*-human share-ome

*Flaviviridae* is a family of small enveloped, positive-stranded RNA viruses of 9000–13,000 bases [69]. Most are pathogenic to humans, other mammals and birds, and are chiefly spread by arthropod vectors (mainly ticks and mosquitoes). The family comprises of over 130 species grouped into four genera (*Flavivirus*, *Hepacivirus*, *Pegivirus*, and *Pestivirus*), with several unclassified species. The diseases associated with the family can range from asymptomatic to symptomatic, including hepatitis (hepaciviruses), haemorrhagic syndromes, fatal mucosal disease (pestiviruses), haemorrhagic fever, encephalitis, and the birth defect microcephaly (Zika virus). The *Flavivirus* genus is the largest with more than 70 viral species, and notably dengue virus alone poses risk to more than 3 billion people [70]. In this study, we applied the approach described herein to define and characterize the *Flaviviridae*-human share-ome. *Flaviviridae* sequences of dengue and West Nile viruses have been previously reported to be shared with other organisms [39, 71], such as mosquito (*Aedes albopictus*), rice (*Oryza sativa* (japonica cultivar-group)) and bacteria (*Chromohalobacter salexigen, Acidiphilium cryptum JF-5, Actinomyces odontolyticus, Burkholderia ambifaria MC40-6, Burkholderia cepacia AMMD,* and *Methylobacterium extorquens PA1*). The results herein provide insights that will help better understand the structure, function and evolution of *Flaviviridae*.

A total of 263,129 *Flaviviridae* and 1,245,872 human protein sequences were retrieved from the NCBI Entrez Protein (nr) database (as of June 2019 and May 2018, respectively) through the NCBI Taxonomy browser (ID: “11050” for *Flaviviridae*) and E-Utilities esearch-efetch (ID: “9606” for human). Removing duplicate sequences by use of the CD-HIT tool [38] filtered out ~ 49% and ~ 76% of the intially retrieved sequences of *Flaviviridae* and human, respectively. The use of the tool *kmerslicer* generated a 9-mer (nonamer) dictionary each from the *Flaviviridae* (134,904) and human (304,430) non-redundant sequences, respectively, resulting in 71,428,325 and 110,721,413 nonamers (redundant dataset). The choice of nonamer (9-mer) size was to balance between random and significant hits.

Removal of duplicate nonamers from each dictionary and cross-matching the remaining nonamers between the dictionaries resulted in a *Flaviviridae*-human share-ome of 2537 nonamers (file size of ~ 1.8 MB) at a threshold of 100% identity. Close inspection of the share-ome revealed that 110 nonamers matched to 79 unique protein records in the human dataset that appeared to be of non-human origin (with the “Organism” field value as “unknown”, “unidentified” or name of a bacterial species). Further inspection revealed that two of the 79 were human sequences, six were chimeric fusion proteins (human and bacteria), and the remaining 71 were of bacterial origin (all were from *Mesorhizobium delmotii*). Such misclassification of protein records was not observed for the *Flaviviridae* dataset. Three of the 110 share-ome nonamers also matched to other human protein sequences, and thus, were retained, while the remaining 107 nonamers, as well as the matched 77 protein records were removed. This resulted in an eventual *Flaviviridae*-human share-ome of 2430 nonamers (file size of ~ 1.8 MB) at a threshold of 100% identity (Supplementary Table 1). Although the share-ome represented a small fraction of the repertoire of *Flaviviridae* (~ 0.12%) and human (~ 0.013%) non-redundant nonamers (2,107,979 and 18,892,169, respectively), the 2430 shared nonamers mapped to 16,946 *Flaviviridae* and 7506 human non-redundant protein sequences. The most abundant shared sequence “PVPPPRKKR” was present in 4268 viral and 13 human protein sequences; while “HHHHHHSSG” was present in 973 human and 10 viral protein sequences. As many as 181 shared sequences were least abundant, each present in only one protein of the virus and the host; this included “AAAAAAGLR” from *Hepacivirus C* “polyprotein” (specifically, from viral RNA dependent RNA polymerase region), shared with the human “EGF-containing fibulin-like extracellular matrix protein 1 isoform X1”, “GENLYFQGM” shared between “Chain E, E Protein” of *Zika virus* and human “Chain A, E3 ubiquitin-protein ligase Mdm2”, and “LGTVAVALG” shared between “polyprotein” of *Theiler’s disease-associated virus* and human “spermatogenesis associated 3, isoform CRA_a” protein, among others. The nonamer “AGCQRVGIS” was multi-species shared across *Bovine viral diarrhea virus 1* (*Pestivirus A*), *Bovine viral diarrhea virus 2* (*Pestivirus B*), and *Bovine viral diarrhea virus 3* (*Pestivirus H*).

The “Second envelope protein (Fragment)” from *Hepacivirus C* was most represented of shared sequences in the *Flaviviridae* proteome, with an SSR of ~ 9.2% (224 shared sequences). Similarly, for the human proteome, the sequence in the protein record “hCG2016179, isoform CRA_f” was the most represented, with an SSR of ~ 8.7% (212 shared sequences); BLAST search revealed that this protein was a homolog to DnaJ protein. In contrast, the least represented proteins of the human and the *Flaviviridae* proteomes totalled to 4448 and 8618, respectively, with just a single shared sequence in each, and thus a negligible SSR of ~ 0.04% for each protein. These proteins included “prostaglandin-endoperoxide synthase-1” and “papillary thyroid carcinoma-encoded protein” for human, among others; while for the *Flaviviridae*, they included “non-structural 2A-(NS2A)-protein” of *Dengue virus 1*, “envelope (E) protein” of *Japanese encephalitis virus*, and “non-structural 4B-(NS4B)-protein” of *GB virus*, among others.

Assessment of SSPs revealed that the “LYST-interacting protein LIP6” in human was possibly the most packed of shared sequences, with an SSP value of ~ 73.8%. The least packed human protein was the “cytochrome P450c17, partial”, among many others, with an SSP value of ~ 1.8%. Similarly, the “polyprotein, partial” of *Bovine viral diarrhea virus 1* was possibly the most packed viral protein, with an SSP value of ~ 33.2% (calculated over the reported length of the partial polyprotein). The “non-structural 2A-(NS2A)-protein” of *Dengue virus 1,* among many others, was the least packed, with an SSP of ~ 0.3%. There were several other hits with an SSP higher than those reported above, but were ignored from SSR, SSP and hotspot evaluations because record metadata review revealed that they were chimeric, synthetic construct, or modified protein (patent sequence). This shows that it may be difficult to remove all irrelevant sequences early on at the “data cleaning and processing” step (see Materials and Methods section). This is because it would require evaluating the meta-data of every single sequence record, coupled with domain expertise for correct interpretation, which can be a challenge for big data. Nonetheless, further checks herein suggested that the number of remaining irrelevant hits from human appeared limited. Moreover, the share-ome nonamers that these irrelevant proteins matched are also anticipated to be matched by other relevant proteins. Thus, the number of share-ome nonamers (2430) was expected to remain the same or change minimally. Separately, it should be noted that the polyprotein matches pose a question of whether to calculate the SSP over the entire length of the polyprotein or to calculate for each of the individual proteins matched within the polyprotein. Additionally, it should be considered that fusion proteins can also be natural, such as those composed of cellular and viral sequences, and thus, should not be filtered.

**Fig. 2:**
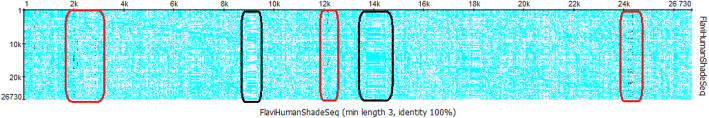
Dot matrix of *Flaviviridae*-human shared sequences at window length of three amino acid residues. Multiple direct repeat regions (cyan areas) were identified in the dot plot. Well-defined regions of low-complexity are outlined in black, while well distinct inverted repeat regions are outlined in dark-red with prominent black dots as the indirect repeats

Analyses for AARs of length three to five amino acids at 100% identity among the 2430 nonamers of the share-ome illustrated innumerable regions of repeats and inverted repeats within and between them (Fig. 2; Table 4; Supplementary Figure 1). Nearly 83% (2026) of the shared sequences formed hotspots in viral proteins (Supplementary Table 2 provides a list of top 200 hotspots), each a cluster of at least three overlapping shared sequences covering a minimum length of 11 amino acids to a large region covering 160 amino acids (aa) of “p125 protein, partial” from *Bovine viral diarrhea virus 1*, with an SSP of ~ 15.2%. Similarly, for human, the longest hotspot covered a region of 95 amino acids, observed in the human protein, “LYST-interacting protein LIP6” (SSP, ~ 73.8%).

**Table 4:** Sample of amino acid repeats (AARs) within the *Flaviviridae*-human shared sequences

Direct Repeat						Indirect Repeat				Simple Repeat	Non- Tandem Repeat (NTR)
3-mer	No. of Matches	4-mer	No. of Matches	5-mer	No. of Matches	3-mer	No. of Matches	4-mer	No. of Matches	9-mer	9-mer
GLL	31	AAAG	14	GGGSG	28	AAA	96	ELKQ	6	GGGGGGGGG	ENVKAKIQD
GVD	17	GGGG	47	GSGGG	27	APA	10	LIKV	3	HHHHHHHHH	ESTLHLVLR
HHH	313	GIPP	13	HHHHH	222	DGK	7				GMQIFVKTL
LLL	293	GRAA	4	LLLLA	18	GLL	31				GRTLSDYNI
LLS	47	HHHH	222	LLLLL	80	LLA	48				HLVLRLRGG
LPP	23	LIGL	2	LLLSL	17	LLL	293				IQKESTLHL
LVL	40	LLGL	19	PNPPKT	4	PLS	16				KESTLHLVL
PPQ	16	LLLL	159	PPPPP	34	SAA	21				LHLVLRLRG
PPR	30	LLSL	26	SHHHH	25	SPR	12				NIQKESTLH
RRL	13	LPVL	10	SSGLV	5	VAA	17				STLHLVLRL

**Fig. 3:**
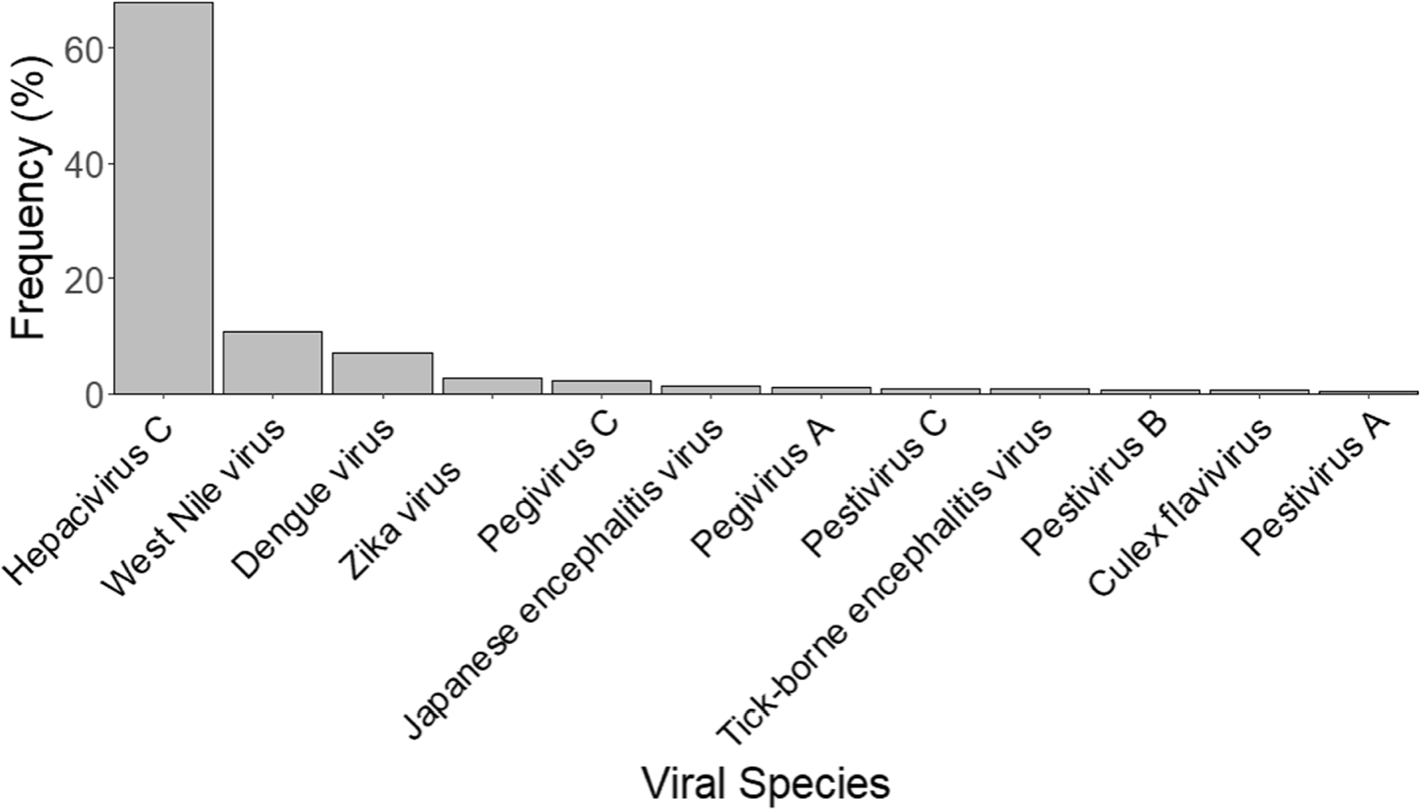
Major *Flaviviridae* species that shared peptides of length nine (100% identical) with human proteins

The shared nonamer sequences mapped to 125 species of *Flaviviridae*, including several with unclassified genus. The majority (~ 68%) of the shared sequences mapped to *Hepacivirus C* species (Fig. 3). The *West Nile, dengue and Zika viruses* of the *Flavivirus* genus accounted for ~ 11%, ~ 7%, and ~ 3%, respectively, of the *Flaviviridae* protein sequences (16,946) mapped by the share-ome. Other notable species or groups of viruses only accounted for less than 1% each, these included: *Tick borne encephalitis virus*, *Japanese encephalitis virus*, *Pestiviruses*, *Pegivirus A*, *Kyasanur forest disease virus, and Yellow fever virus,* among others. The *Hepacivirus C* and *Dengue viruses* also had their various genotype sequences well represented in the share-ome. *Hepatitis C virus genotype 1* accounted for ~ 33% of the ~ 68% *Hepacivirus C* shared sequences in the share-ome, while *Dengue virus serotype 1* accounted for ~ 41% of the ~ 7% of *Dengue viruses*.

**Fig. 4:**
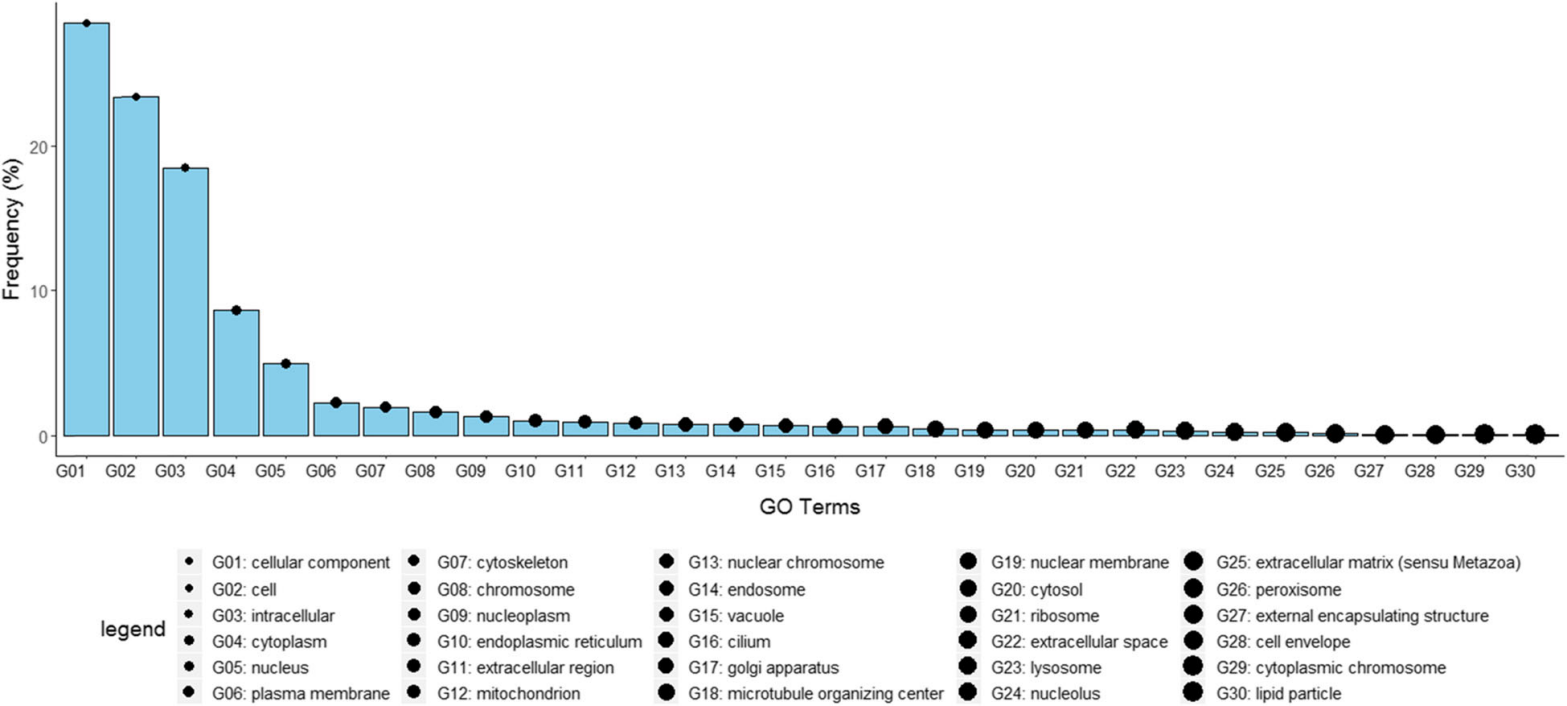
Cellular localization of the human proteins that contained the *Flaviviridae*-human shared sequences

Gene ontologies of the shared sequence were evaluated from the 7506 human protein (IDs) identified for the share-ome, which mapped to 2376 gene names in UniProt. However, for enrichment analysis, only 1132 records for cellular compartment GO Terms were annotated and retrieved for analysis. The 1132 GO terms were reduced mapped to 127 “GO_slim” terms (Fig. 4) by use of a GO Terms Classification Counter – CateGOrizer [72]. Notably, the high frequency terms implied localization of the proteins in the nucleus, plasma membrane and cytoskeleton. Studies have shown that non-structural protein 3 (NS3) and NS5 of flaviruses interact with the cellular component cytoskeleton in human [73].

**Fig. 5:**
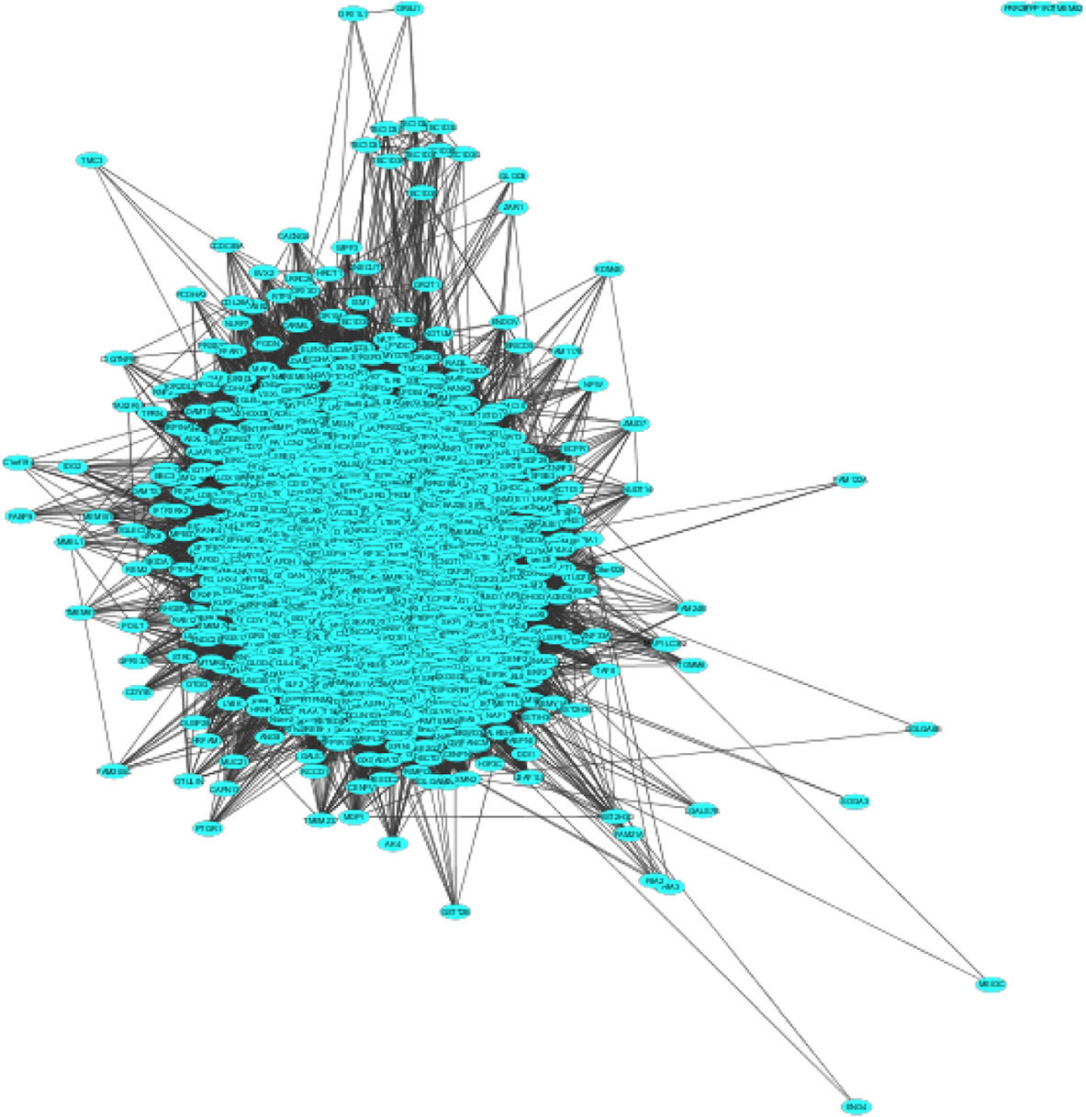
*Flaviviridae*-human share-ome interaction network of 2001 nodes and 215,897 edges. The top 20 human, hub genes with node degree of 300 and above for the *Flaviviridae*-human share-ome interaction network are shown in Table 5

Biological process involvement of the 2376 human genes was evaluated by use of GeneMANIA Cytoscape plugin, which produced 2001 nodes and 215,897 edges (setting “biological processes” for the Gene Ontology term and “*Homo sapiens*” for source species) (Fig. 5). The most enriched biological process terms in the network were metabolism, cell communication and signal transduction, among others (data not shown). Network analyses of the nodes using the Network Analyzer of Cytoscape predicted 1879 hub genes, with node degree of 50 and above. The gene Ubiquitin C (UBC) had the highest node degree of 1441 (Table 5) and it is reported to code for “polyubiquitin-C” protein [74]. Polyubiquitin-C is one of the sources of maintaining the ubiquitin homeostasis state under normal physiological conditions [75]. It also plays a key role in sustaining responses associated to UV irradiation, heat shock, oxidative stress and translational impairment. In addition, UBC being the gene with the highest node degree, where it interacts with a large population of genes, suggesting its importance as a possible share-ome mediator. The Ubiquitin (Ub) protein can be conjugated to select proteins to modulate their turnover and signalling. The protein Ub’s broad role in cellular processes, such as protein trafficking, cell-cycle regulation, DNA repair, apoptosis and signal transduction, among others [75], have important clinical implications.

**Table 5:** Top 20 human, hub genes with node degree of 300 and above for the *Flaviviridae*-human share-ome interaction network

Gene name	Degree	Betweenness Centrality	Closeness Centrality	Clustering Coefficient
UBC	1441	0.027056	0.781299	0.139379
APP	665	0.004432	0.59898	0.166061
FN1	645	0.003141	0.595586	0.184236
XPO1	610	0.002835	0.588738	0.220372
AUTS2	606	0.002965	0.588912	0.181431
SRPK1	605	0.00213	0.588218	0.218477
CAND1	601	0.0021	0.585802	0.204786
CELF2	593	0.002288	0.58649	0.204714
TRIM28	579	0.002369	0.583236	0.199306
RBFOX1	576	0.002589	0.583577	0.18808
DAPK1	566	0.002433	0.581538	0.187911
PIK3R1	553	0.002085	0.578505	0.198063
DLG2	552	0.002489	0.577836	0.186907
EGFR	551	0.002312	0.578673	0.186986
PRKG1	527	0.001923	0.575339	0.187856
SUMO1	526	0.001842	0.574676	0.221619
PPP2CA	525	0.001574	0.574676	0.225096
IQGAP2	519	0.001925	0.573851	0.183431
EPS15	519	0.001614	0.571551	0.206002
KDM1A	516	0.002118	0.571224	0.186227

**Fig. 6:**
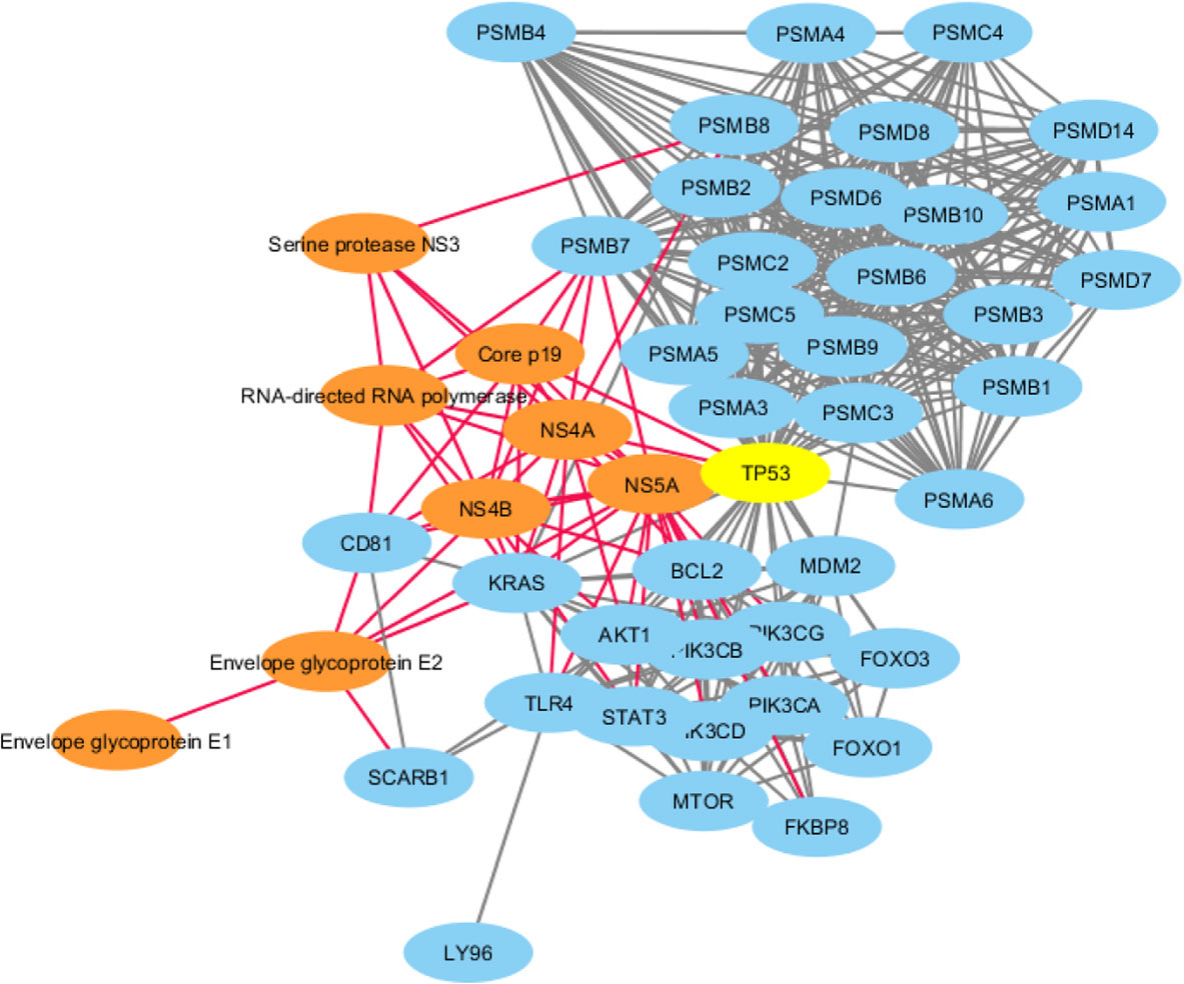
*Hepatitis C virus* (HCV) *genotype 1a-*human protein-protein interaction (PPI) network. The HCV proteins associated with major hub, human proteins (TP53, PSMB7, and PSMB8, among others; Table 6). Orange nodes denote viral proteins with red edges linking to other nodes; blue nodes denote human proteins with grey edges linking to various nodes; and the yellow node denotes the hub protein with the highest degree of nodes. The TP53 is a hub connecting major nodes of the HCV genotype 1a-human PPI network

**Fig. 7:**
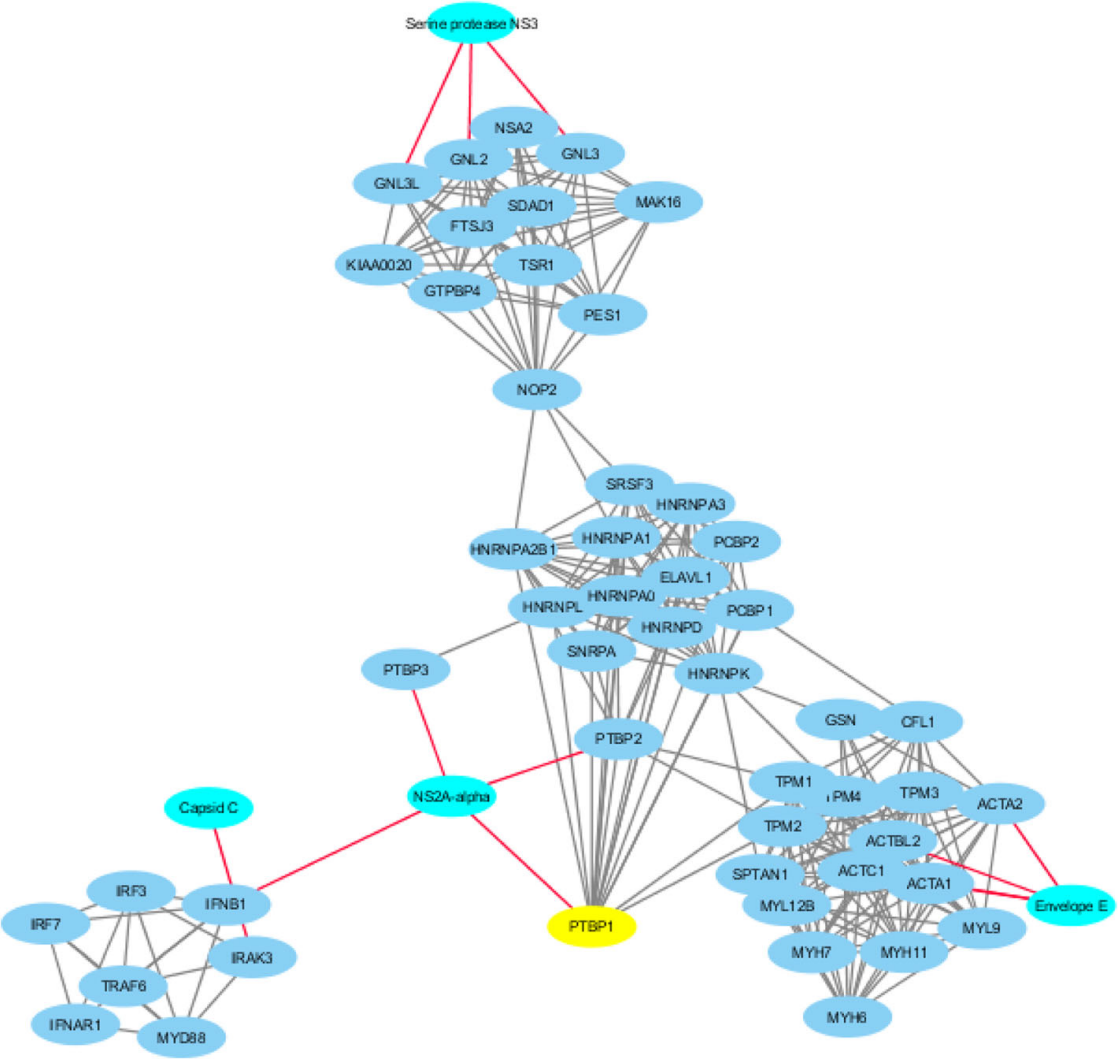
*Dengue virus type* (DV) *2* (strain Jamaica/1409/1983)*-*human protein-protein interaction (PPI) network. The DV proteins associated with major hub, human proteins (PTBP1, ACTC1 and ACTA2, among others; Table 7). Cyan nodes denote viral proteins with red edges linking to other nodes; blue nodes denote human proteins with grey edges linking to various nodes; and the yellow node denotes the hub protein with the highest degree of nodes. The PTBP1 is a hub connecting major nodes, including NS2A-alpha, which connects other viral and human nodes to PTBP1 and to other nodes of the DV-human PPI network

The *Flaviviridae*-human share-ome was functionally analysed for viral-host protein-protein interactions (PPI). The top 200 hotspot containing viral protein sequences (Supplementary Table 2) were subjected to this interaction analysis. Only 24 of these viral proteins were mapped to unique UniProt IDs, which were submitted to STRING Viruses database (Table 1) [76] for PPI. This returned a match to 10 viral organisms, two of which were further studied (match to Hepatitis C virus (HCV) genotype 1a (isolate H) and Dengue virus (DV) type 2 (strain Jamaica/1409/1983)) as representative results of the analysis. *Flaviviridae*-human protein-protein interaction network for *Hepatitis C virus genotype 1a* (Fig. 6) and *Dengue virus type 2* (strain Jamaica/1409/1983) (Fig. 7) revealed eight and four viral proteins, respectively, involved in the interaction with human proteins. In the case of HCV genotype 1a, the eight viral proteins were “NS4A”, “RNA-directed RNA polymerase”, “Core p19”, “Serine protease”, “NS5A”, “NS4B”, “Envelope glycoprotein E2” and “Envelope glycoprotein E1”, which were involved in the PPI with over 40 human proteins, directly or indirectly. The human TP53, which contained at least a shared sequence, was a hub protein with the highest node degree (Table 6). TP53, a “Cellular tumor antigen p53” protein, functions as a tumor suppressor in various types of tumors; regulates growth or apoptosis depending on the physiological conditions and cell type, among others [77]. The HCV NS5A, a non-structural protein occurs in two forms (p56 and p58), before being activated and released as NS5A and then localizes in the nuclear periplasmic membrane [78]. Notably, studies have shown association between NS5A and TP53, where the interaction appears to lead to transcriptional modulation of the p21/waf1 gene and is suggested to be one of the contributory factors towards HCV-mediated pathogenesis. In the case of DV type 2 (strain Jamaica/1409/1983)-human PPI network (Fig. 7), the four viral proteins “Capsid C”, “NS2A-alpha”, “Envelope E”, and “Serine protease NS3” were involved in the PPI with 43 human proteins. The human protein PTBP1, which contained at least a shared sequence, was a hub protein with the highest node degree (Table 7). PTBP1, a “Polypyrimidine tract-binding protein 1”, functions as a regulating protein involved in mRNA splicing, especially in muscle cell differentiation. The polypyrimidine tract-binding protein (PTB) interacts with the regulatory sequences of positive-strand RNA viruses in the cytoplasm; PTB is translocated from the nucleus to the cytoplasm during DV infection [79]. Post infection, NS1 and NS3 of DV and human PTB are observed to co-localize with the endoplasmic reticulum marker calnexin. It has been shown that when PTB is not expressed, DV translation and replication are inhibited; PTB expression facilitates viral propagation. This suggests PTB and DV protein PPI in cytoplasmic environment plays a key role in dengue pathogenesis.

**Table 6:** Top 10 hub genes with node degree of 22 and above for the *Hepatitis C virus (HCV) genotype 1a*-human protein-protein interaction network

Gene Name	Degree	Betweenness Centrality	Closeness Centrality	Number of Directed Edges
TP53	39	0.34606769	0.83928571	39
PSMB7	27	0.07554848	0.67142857	27
PSMB8	25	0.04383678	0.64383562	25
PSMC3	23	0.00613026	0.6025641	23
PSMA3	23	0.00613026	0.6025641	23
PSMB3	22	0	0.59493671	22
PSMB10	22	0	0.59493671	22
PSMA4	22	0	0.59493671	22
PSMD14	22	0	0.59493671	22
PSMB4	22	0	0.59493671	22

**Table 7:** Top 10 hub genes with node degree of 14 and above for *Dengue virus type (DV) 2 (strain Jamaica/1409/1983)*-human protein-protein interaction network

Gene Name	Degree	Betweenness Centrality	Closeness Centrality	Number of Directed Edges
PTBP1	16	0.26321534	0.46491228	16
TPM2	16	0.10688075	0.39259259	16
TPM4	16	0.03998649	0.36805556	16
TPM1	16	0.10688075	0.39259259	16
HNRNPK	15	0.13451659	0.43089431	15
HNRNPA1	14	0.1326909	0.43089431	14
HNRNPA2B1	14	0.1326909	0.43089431	14
ACTC1	14	0.01092874	0.31176471	14
TPM3	14	0.00105224	0.31176471	14
NOP2	14	0.35703919	0.3557047	14

Additional literature mining for functional characterization of *Flaviviridae*-human shared sequences was carried out. Penta- and hexa-peptide (5- and 6-mer) sharing between Zika virus (ZIKV, a fetopathogen and microcephaly-associated) polyprotein and human proteins linked/related to brain calcification, myelin, (de)myelination, and/or axonal neuropathies have been reported, and a number of the peptides were experimentally validated as immunopositive epitopes in human [80]. The study suggested cross-reactivity mechanism as a link between the infection and brain damage/neurodevelopmental disturbances observed in infants, as well as individuals with Guillain-Barré-like syndromes. Given that the search for longer shared sequences may miss shorter ones, a few of the peptides identified by [80] remained shared at nonamer level as part of the *Flaviviridae*-human share-ome identified herein. For example, the pentapeptide LGLTA in VAALGLTAV was shared between polyprotein of Zika virus and human “MOCO sulphurase C-terminal domain containing 1 (also known Mitochondrial amidoxime-reducing component 1)”, “mitochondrial amidoxime-reducing component 1 precursor” and “mitochondrial amidoxime-reducing component 1 isoform X1” proteins (MOSC1). This MOSC1 is similar to human “Sulfite oxidase, mitochondrial”, one of the microcephaly-related human proteins [81]. Another, pentapeptide LLGLL was observed in validated epitopes by [82], such as in RLLLLGLLLL and FLLGLLFFV, among others, which originated from viral proteins associated with autoimmune reactions, of the following fetopathogens: ZIKV, *Human cytomegalovirus* (HCMV), and *Toxoplasma gondi* [82]. Several additional nonapeptides (9-mers) containing the LLGLL motif were identified, all of which were observed in *Hepacivirus C* (hepatitis C virus; HCV) “NS5” RNA-dependent RNA polymerase and shared with 20 human proteins, which included “chordin protein” and “desmoglein 3 (pemphigus vulgaris antigen), isoform CRA_a”, among others. The earlier study [82] did not identify any shared penta- or hexa-peptide from *Hepacivirus C*, perhaps because they only studied 11 proteins of this virus (HCV) and restricted the search to human microcephaly-related proteins only, whereas herein all reported *Flaviviridae* and human protein sequences were compared. A literature search for “*Hepacivirus C* and fetopathogenesis” was to no avail, and thus, this shared sequence match suggested a different role of the nonapeptide compared to the shorter penta- and hexapeptides that matched to human microcephaly-related proteins.

## Discussion

Shared sequences represent a multi-faceted key to understanding the host-pathogen interactome, from functional, structural, evolutionary, and immunological perspectives [21, 83, 84]. Pathogens can interfere with the normal biological processes of their host, specifically by targeting the cell component, metabolism and/or metabolite [85], among others, facilitated by shared sequences. The bioinformatics approach presented herein provides a workflow and considerations to identify and characterize the host-pathogen share-ome, taking advantage of the big data in public repositories. Earlier studies were limited to identifying shared sequences for specific pathogen species of interest. While the workflow herein is essentially similar to the various works by others [16, 17, 21, 23, 25], the generic methodology is described systematically, including details and various considerations, while providing additional new dimensions to certain aspects of the workflow. The current workflow was designed around the research goal of mapping and characterizing the share-ome. Comparison was done with the existing approaches, such as Peptide Match [86, 87], and where applicable, a similar strategy was applied, and new ones developed where lacking. This was particularly so in the area of structural-functional characterizations. As for mapping, the difference with earlier workflow was largely in the size of the *k-mer* utilised and also the scale of the sequences analysed. Taken together, this resulted in a new workflow, which enables a comprehensive and exhaustive mapping of shared sequences at big data scale between all reported pathogen sequences, at any given rank of taxonomy lineage, and all reported host sequences, at a chosen taxonomic rank. It is hoped that the work herein would facilitate other share-ome studies to be carried out rapidly, enabling comparative share-ome analyses.

Mapping of the share-ome has important implications towards the design of vaccines and drugs, and development of surveillance and diagnostic strategies against pathogens. Shared sequences predicted and/or validated to be immune relevant (e.g. as B/T-cell epitopes) may need to be filtered from inclusion as vaccine targets since they are shared with the host proteome [88]. Such sequences may escape immune recognition as a self-antigen or elicit an autoimmune response within the host through molecular mimicry [89–91]. A catalogue of the shared sequences can act as a reference for researchers involved in vaccine design. As for drug design, shared sequences are likely to be functionally and structurally important [92] and thus, expected to be evolutionarily conserved; therefore, inhibitory ligands may be designed against them to block pathogen activity. However, potential for side effect exists, given that they are also present in the host proteome. Thus, in general, shared sequences may be avoided from use as targets for inhibitory drug design. Nonetheless, careful evaluation of the pathogen and host proteins containing the shared sequence may offer insights on structural differences between them, which may abrogate the inhibitory ligand binding with the host protein, while being effective against the pathogen. Additionally, even if ligand binding ability is congruent with proteins of both pathogen and the host, there may be differences in the bioavailability of the host protein [93], and thus, reducing the possibility of side effects. Shared sequences may not meet the basic definition of a candidate diagnostic target, which are preferred to be conserved and specific to the organism of interest. Shared sequences may be conserved, but may not be specific; also, the level of conservation may not be at the desired threshold. Even if they meet the desired criteria for use as a diagnostic target, care must be taken to discriminate a false positive match with the host sequences, originating from remnant host cells in the sample. A catalogue of species relevant to a host-pathogen share-ome of interest, such as a list of all *Flaviviridae* species that share sequences with human, may reveal those that are not yet recognised as a threat to the host. Such species could be further evaluated for inclusion as candidates for surveillance under the list of emerging or priority pathogens. Additionally, ascertaining those that are known and medically important from the share-ome species catalogue may provide additional dimension to re-evaluate an existing priority pathogen list (PPL) [94, 95].

Herein, we described a systematic bioinformatics approach for identification and characterization of shared sequences from big data. Some of the big data challenges that may be encountered in executing the workflow include data download time (even with NCBI Entrez E-utilities API), deduplication of the data by use of CD-HIT, dissection of the sequences into *k-mers* to generate the dictionaries, identification of AARs by use of UGENE, and mapping of the hotspots, among others. Resolution of these challenges include a combination of approaches, such as download of data when the bandwidth may be lesser occupied, such as over weekends, or getting access to a dedicated bandwidth, such as by use of a national Research Education Network (REN); a “break-and-conquer” approach by splitting the FASTA sequence file to various sizes to handle “out of memory” issues or by allocating a larger memory resource, or a combination of both. A cloud platform may be desired when access to an in-house high-performance computing is limited or is unavailable, provided a budget is at disposal to pay for the run-time; though the cost may be reasonable as it can be usage dependent. A workflow that is well-defined helps plan for these challenges accordingly and identify appropriate solutions, enabling a focus on the myriad of research questions possible from the data.

## Conclusion

The workflow herein is generic and applicable to a broad variety of pathogens, such as viruses, bacteria, parasites, among others. The methodology significantly expands the breadth and depth of existing approaches. It enables the systematic screening and characterization of pathogen and host data which would otherwise be impossible to carry out experimentally, due to too many pathogen sequences (high pathogen diversity) and the large repertoire of the host proteome. It therefore significantly reduces the efforts and cost of experimentation, while providing for systematic screening. The *Flaviviridae*-human share-ome provided important structural and functional insights that help better understand the host-pathogen interaction of this important family of viruses, which poses an expanding threat to public health. 

## Supplementary Information

**Additional file 1: Supplementary Figure 1.** Dot matrix of *Flaviviridae*-human shared sequences at window lengths of three (A), four (B), and five (C) amino acid residues. Multiple direct repeat regions (cyan areas) were identified in all the dot plots. A) and B) show well-defined regions of low-complexity (outlined in black). Inverted repeats are well distinct in (A and B) (regions outlined in dark-red with prominent black dots as the indirect repeats).

**Additional file 2: Supplementary Table 1.** A catalogue of *Flaviviridae*-Human share-ome nonapeptide sequences.

**Additional file 3: Supplementary Table 2.** Major clusters (hotspots) of *Flaviviridae*-Human shared sequences in proteins of *Flaviviridae* family viruses. This list includes Protein ID that may be chimeric, synthetic construct, or modified protein (patent sequence).

## Data Availability

The datasets analysed herein are available at https://www.ncbi.nlm.nih.gov/protein/?term=txid11050[Organism:exp] and https://www.ncbi.nlm.nih.gov/protein/?term=txid9606[Organism:noexp] for *Flaviviridae and* human proteomes, respectively. The data that support the findings of this study are available here: https://github.com/gwatiyapJ/shareomeHF

## Abbreviations

aa: Amino acids
AARs: Amino acid repeats
API: Application programme interface
CDD: Conserved domain database
CD-HIT: Cluster Database at High Identity with Tolerance
CMV: Cytomegalovirus
DV: Dengue virus
GO: Gene Ontology
HCMV: Human cytomegalovirus
HCV: Hepatitis C virus (*Hepacivirus C*)
HIV-1: Human immunodeficiency virus 1
HLA: Human leukocyte antigen
ID: Identification number
NCBI: National Centre for Biotechnology Information
NS: Non-structural protein
PDB: Protein Data Bank
Pfam: Protein family database
PPI: Protein-protein interaction
PPL: Priority pathogen list
REN: Research Education Network
RNA: Ribonucleic acid
SSP: Shared sequence packing
SSR: Shared sequence representation
UBC: Ubiquitin C
URL: Uniform Resource Locator
WNV: West Nile virus
ZIKV: Zika virus
