# Supplementary material for: A systematic bioinformatics approach for large-scale identification and characterization of host-pathogen shared sequences
Source: BMC Genomics. 2021 Sep 28;22(Suppl 3):700. doi: 10.1186/s12864-021-07657-4 (PMC8477458; doi:10.1186/s12864-021-07657-4)
Supplement: Supplementary file 1 — Additional file 1: Supplementary Figure 1. Dot matrix of Flaviviridae-human shared sequences at window lengths of three (A), four (B), and five (C) amino acid residues. Multiple direct repeat regions (cyan areas) were identified in all the dot plots. A) and B) show well-defined regions of low-complexity (outlined in black). Inverted repeats are well distinct in (A and B) (regions outlined in dark-red with prominent black dots as the indirect repeats). [file 12864_2021_7657_MOESM1_ESM.docx]

**Supplementary Figure 1**

A)

**
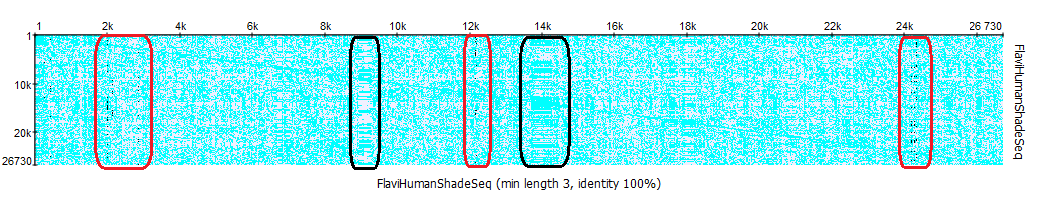
**

B)

**
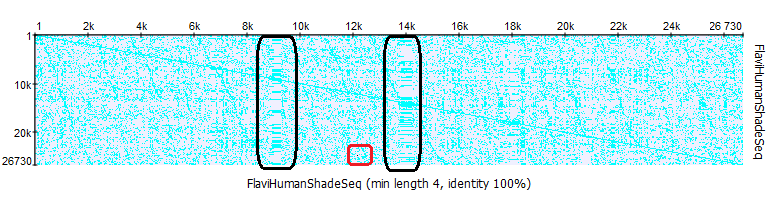
**

C)

**
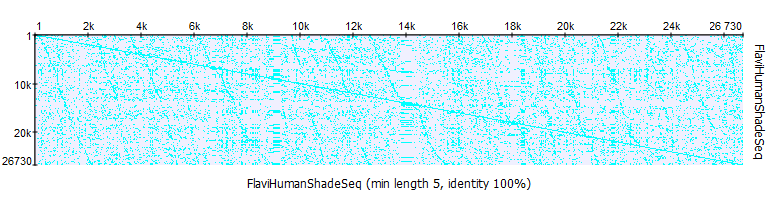
**

**Supplementary Figure 1:** Dot matrix of *Flaviviridae*-human shared sequences at window lengths of three (A), four (B), and five (C) amino acid residues. Multiple direct repeat regions (cyan areas) were identified in all the dot plots. A) and B) show well-defined regions of low-complexity (outlined in black). Inverted repeats are well distinct in (A and B) (regions outlined in dark red with prominent black dots as the indirect repeats)
